# Probing Heme Active Sites of Hemoglobin in Functional Red Blood Cells Using Resonance Raman Spectroscopy

**DOI:** 10.1021/acs.jpcb.1c01199

**Published:** 2021-03-31

**Authors:** Jakub Dybas, Tapiwa Chiura, Katarzyna M. Marzec, Piotr J. Mak

**Affiliations:** † Chemistry Department, Saint Louis University, 3501 Laclede Avenue, Saint Louis 63103, Missouri, United States; ‡ Jagiellonian Centre for Experimental Therapeutics (JCET), Jagiellonian University, 14 Bobrzyńskiego Str., Krakow 30-348, Poland

## Abstract

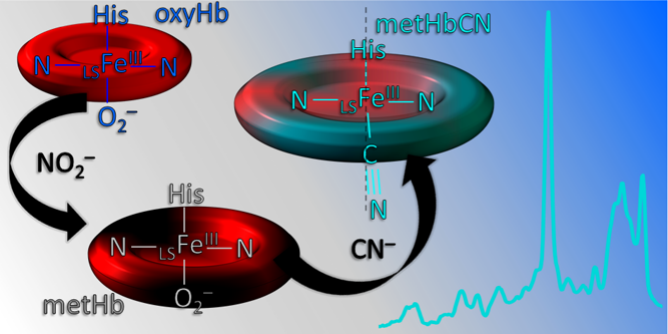

The UV–vis absorption, Raman imaging, and resonance Raman (rR) spectroscopy methods were employed to study cyanohemoglobin (HbCN) adducts inside living functional red blood cells (RBCs). The cyanide ligands are especially optically sensitive probes of the active site environment of heme proteins. The rR studies of HbCN and its isotopic analogues (^13^CN^–^, C^15^N^–^, and ^13^C^15^N^–^), as well as a careful deconvolution of spectral data, revealed that the ν(Fe–CN) stretching, δ(Fe–CN) bending, and ν(C≡N) stretching modes occur at 454, 382, and 2123 cm^–1^, respectively. Interestingly, while the ν(Fe–CN) modes exhibit the same frequencies in both the isolated and RBC-enclosed hemoglobin molecules, small frequency differences are observed in the δ(Fe–CN) bending modes and the values of their isotopic shifts. These studies show that even though the overall tilted conformation of the Fe–C≡N fragment in the isolated HbCN is preserved in the HbCN enclosed within living cells, there is a small difference in the degree of distortion of the Fe–C≡N fragment. The slight changes in the ligand geometry can be reasonably attributed to the high ordering and tight packing of Hb molecules inside RBCs.

## Introduction

Cyanohemoglobin (HbCN) is a hemoglobin (Hb) adduct containing a low-spin (LS) ferric heme with a bonded cyanide ion (CN^–^).^1−6^ This adduct is often considered to be the main reason for severe hypoxia and eventually death during cyanide poisoning;^7,8^ however, it is not more toxic than methemoglobin (metHb) itself; it is yet another form of physiologically nonfunctional ferric Hb, unable to bind and transport oxygen molecules.^9^ Due to its stability, HbCN adducts are often used in the determination of the total Hb concentration in the blood using absorption spectroscopy.^10,11^ In the absence of any steric hindrance, as in the case of free iron protoporphyrin IX, cyanide anion binds to ferric heme in a strictly linear geometry, perpendicular to the heme macrocycle plane,^12,13^ similar to the ferrous CO adducts.^12^ However, in heme proteins, the CN^–^ ligand interacts with the active site amino acid residues, and the presence of steric or polar interactions can induce a conformational change of the Fe–C≡N fragment.^14,15^ The Fe–C≡N linkage can adopt tilted linear (also called “essentially linear”) or bent geometries, depending on the nature of steric and/or H-bonding interactions.^14−16^ The high sensitivity and responsiveness of the Fe–C≡N linkage to the heme pocket environment make cyanide ligands an excellent probe of the active site structure.^14^ Structural insights into the heme pocket environment and factors that can change its architecture are essential for better understanding the structure–function correlation in heme proteins.

The X-ray studies of a relaxed quaternary structure of Hb showed that there is not enough space in the distal heme pocket for a sterically unhindered linear configuration along the heme axis.^17^ Therefore, it was suggested that the Fe–C≡N fragment actually remains linear but assumes a tilted geometry with approximately 20% displacement from the heme normal.^12,14,17^ Such changes in the Fe–C≡N configuration lead to an overlap between π* porphyrin and cyanide orbitals, leading to a coupling of the Fe–C≡N bending mode with the resonant Soret (π → π*) transition,^15^ and consequently allow the enhancement of the heme–CN^–^ vibrational modes. Resonance Raman (rR) spectroscopy is a powerful technique for the characterization of the heme–CN^–^ adducts, allowing the detection and monitoring of all possible vibrations associated with the Fe–C≡N fragment, including the ν(Fe–CN) stretching, δ(Fe–CN) bending, and ν(C≡N) stretching modes;^12,14,15,18−20^ for example, the rR studies of isolated HbCN protein were previously published.^1,5,12^

The enhancement of specific vibrational modes, their frequencies, and patterns of isotopic shifts permit a clear distinction between the linear, tilted, and bent configurations of the Fe–C≡N fragment. The linear geometry of the Fe–CN linkage naturally abolishes the activation of the δ(Fe–CN) bending mode.^14,18^ In cases when the active site perturbs the linkage sufficiently strong, the Fe–C≡N unit will adopt a tilted (essentially linear) or bent configuration, for which the δ(Fe–CN) bending mode is effectively enhanced.^14,18^ In some heme proteins, such as Mb, Hb, and cytochrome *c* oxidase (CcO), the active site exerts only a slight disturbance of the axial ligand, resulting in the tilted configuration of Fe–C≡N. For a tilted conformer, the ν(Fe–CN) stretching mode is usually seen at around 450 cm^–1^, whereas the δ(Fe–CN) bending mode is seen around 380 cm^–1^.^12,14,15^ Analysis of the rR spectra of isotopically substituted analogues provides additional information regarding the correct assignment of the Fe–C≡N geometry. In the case of the tilted conformer, the ν(Fe–CN) stretching mode decreases in a monotonic manner with the increase in the total mass of CN^–^ in the following order: CN, ^13^CN, C^15^N, ^13^C^15^N. On the other hand, the δ(Fe–CN) bending modes of the tilted conformer exhibits the so-called zig-zag pattern, where the significant frequency decrease is observed only for the ^13^C isotopologues.^12,16^ If active site perturbations are sufficiently strong, as in the case of lactoperoxidase (LPO) and myeloperoxidase (MPO) proteins, they can induce a substantial degree of distortion, resulting in a bent configuration of Fe–C≡N with a distinct vibrational behavior. The ν(Fe–CN) stretching mode is seen at lower frequencies and exhibits an isotopic zig-zag pattern, whereas the δ(Fe–CN) bending mode is observed at a higher frequency, and its isotopic shifts are monotonic with an increased CN^–^ mass.^14^ It is also noted that in some proteins, such as HRP and P450, the active site architecture allows the coexistence of both the essentially linear and the bent conformers of Fe–C≡N.^14^

Although all previous studies of the CN adducts were done using isolated heme proteins or standard compounds, for example, hemoglobin and myoglobin,^1,12^ cytochrome *c* oxidase,^12,16^ and cytochrome P450,^14,18^ the goal of this work is to probe cyanide ligand binding to Hb inside living red blood cells (RBCs) in order to compare the heme active site properties between isolated and RBC-enclosed Hb molecules. It was previously demonstrated that Raman spectroscopy (RS) is extremely useful in studying Hb alterations within functional RBCs even on a single-cell level and it is an excellent tool to study the heme active site.^21−27^ Such structural information is not available using traditional techniques, including X-ray crystallography, electron microscopy, or NMR spectroscopy. Similarly, molecular modeling calculations are not capable to deliver such insights into proteins encapsulated within RBCs, as a correct assumption of all intercellular interactions would be too overparameterized. Therefore, rR spectroscopy remains unique in assessing the structural information on proteins in complex biological matrixes, and in this work is used for the first time to characterize HbCN adducts formed inside functional RBCs. The work presented herein is complemented by simultaneous rR studies of isolated HbCN proteins in buffer solution. The data provide new evidence that studies of isolated proteins can generally be translated into physiological cell-based investigations. However, some subtle differences are noted between the active site structures of isolated proteins and those within living cells. The studies shown here are a continuation of previous works on RBCs, where the formation of heme adducts in various experimental conditions was studied using RS.^22−25,28^ The spectroscopic studies of HbCN adducts formed inside RBCs were conducted using UV–vis absorption, Raman imaging (RI), and rR spectroscopy. The isotope-sensitive experiments with the use of ^13^CN^–^, C^15^N^–^, and ^13^C^15^N^–^ analogues, as well as careful deconvolution of spectral data, were carried out to derive the frequencies of the modes associated with the Fe–C≡N fragments and to detect the correct values of their isotopic shifts.

## Materials and Methods

### Chemicals and Solutions

Sodium chloride was purchased from Fisher Scientific (Hampton, New Hampshire, USA) and sucrose from Bio Basic (Markham ON, Canada). Sodium nitrite, sodium dithionite, potassium ferricyanide(III), and potassium cyanide were purchased from Sigma-Aldrich (St. Louis, Missouri, USA), whereas the isotopic derivatives of the latter—KC^13^N, KC^15^N and K^13^C^15^N—were purchased from Cambridge Isotope Laboratories (Tewksbury, Massachusetts, USA).

RBCs were washed and stored in 0.9% sodium chloride solution supplemented with 0.2% sucrose, and the pH was adjusted to 7.4 using 1 M hydrochloric acid in order to keep the cells in functional conditions, as previously published.^21^

The Ringer-Tris buffer solution used to wash RBCs was prepared *ex tempore*, as described previously,^22,23^ with the following composition: 140.5 mM NaCl, 2 mM CaCl_2_, 4.7 mM KCl, 1.2 mM MgSO_4_, 21 mM Tris base, 5.5 mM glucose, and 76 μM bovine albumin. All reagents were dissolved in distilled water and filtered through a 0.22 μm pleated filter; the pH was adjusted to 7.40 using 1 M hydrochloric acid. All the above-mentioned chemicals were purchased from Sigma-Aldrich.

### Sample Preparation

#### Red Blood Cells for the rR and UV–vis Measurements

Human RBCs were purchased from the Interstate Blood Bank, Inc. (Memphis, Tennessee, USA). The RBC samples were washed by a triple centrifugation process at 800*g*, 4 °C, with 0.9% sodium chloride solution supplemented with 0.2% sucrose and the pH adjusted to 7.4. The supernatant and the buffy coat were removed by aspiration (after each spin). RBCs were suspended in the same solution in hematocrit (Hct), that is, the volume percentage of RBCs in blood or solution, approximately 0.1% (what corresponds to about 5 μM of Hb and 20 μM of heme).

#### Isolation of Human Hb for rR and UV–vis Measurements

RBCs were diluted 1:1 (v/v) with 0.9% solution of NaCl and subjected to triple centrifugation (8000*g* for 15 min at 4 °C), followed by the removal of the supernatant and buffy coat each time. After third centrifugation, the RBCs were diluted 1:4 (v/v) with cold deionized water (4 °C), and the samples were then stored for 30 min at 4 °C to allow cell lysis. The lysed cells were then subjected to final centrifugation at an acceleration of 23,000*g* for 90 min at 4 °C. The supernatant of the isolated Hb was aspirated and stored at 4 °C. The concentration of Hb in the oxygenated form (oxyHb) was determined using UV–vis absorption spectroscopy. The protein concentrations for rR and UV–vis measurements were approximately 12.5 μM in the Hb tetramer or 50 μM in heme.

#### RBCs for RI

The RBC samples for RI measurements were prepared as described previously.^22,23^ Briefly, human blood samples were collected on heparin as an anticoagulant from healthy volunteers on the day of the experiment. Whole blood samples were subjected to a gentle triple centrifugation process to avoid any membrane damage (acceleration: 500*g* for 10 min at 21 °C). The supernatant and the buffy coat were removed by aspiration after each spin, and the RBCs were washed using a Ringer-Tris buffer solution supplemented with bovine albumin and glucose. RI was conducted within 8 h after collecting the blood samples. RBCs were diluted to approximately 0.1% Hct with the Ringer-Tris buffer solution and transferred to a glass-bottom dish with a CaF_2_ slide.

#### Preparation of HbCN Adducts

The majority of hemoglobin inside RBCs is in an oxygenated form, which is oxyhemoglobin (oxyHb). In order to prepare ferric CN adducts of hemoglobin within an RBC, or in its isolated state, the protein was first oxidized to a ferric state and then treated with an excess of the corresponding KCN salt. oxyHb was reduced anaerobically to deoxyhemoglobin (deoxyHb) using a 10-fold molar excess of freshly prepared sodium dithionite, followed by treatment with NaNO_2_ (10 mM) for 15 min at room temperature, to obtain metHb within functional RBCs. The excess of NaNO_2_ was then removed from the samples by buffer exchange. The samples of isolated oxyHb were incubated with 1.2 M excess of potassium ferricyanide(III) to form isolated metHb, followed by purification using size-exclusion chromatography (Bio-Gel P-6, BioRad). The purity of isolated and RBC-enclosed metHb molecules was confirmed by their characteristic spectral profiles in rR and UV–vis measurements.^21^ To prepare HbCN adducts and its isotopic substituents, samples of metHb were treated with a 10-fold molar excess of KCN, K^13^CN, KC^15^N, and K^13^C^15^N with respect to the heme concentration. The cyanide stock solutions of the respective KCN salts were freshly prepared in water alkalized with NaOH to pH 9. The cyanide excess was not removed from the samples and was present during all measurements. The pH of the samples increased from 7.4 to 7.8 in the presence of cyanides.

### Data Acquisition and Analysis

#### rR Spectroscopy

The rR spectra were recorded using the 406.7 nm excitation line produced by Innova 302C Kr^+^ laser (Coherent Inc., Santa Clara, California, USA) and collected using a 1250M-Series II high-resolution spectrometer with 1250 mm focal length (Horiba Ltd., Kyoto, Japan), equipped with a liquid nitrogen-cooled PyLoN:400B CCD detector (Princeton Instrument, Trenton, New Jersey, USA). Measurements were done using a 180° backscattering geometry, and the laser beam was focused onto the sample using a cylindrical lens. The laser power at the sample was adjusted to approximately 5 mW. The spectral resolution was equal to 1.5 cm^–1^. The 5 mm diameter NMR sample tubes were spun to avoid local heating and ligand photodissociation. All measurements were conducted at room temperature. The slit width was set at 150 μm, and 1200 g/mm grating was used. The spectra were calibrated using fenchone and acetone-*d*_6_ (Sigma-Aldrich, St. Louis, Missouri, USA) and processed with Grams/32 AI software (Galactic Industries, Salem, New Hampshire, USA) and OriginPro 2018 (OriginLab, Northampton, Massachusetts, USA). The spectra were postprocessed (cosmic spike removal was with a median filter of 3 × 3 and background subtraction with the asymmetric least-squares method) and normalized using *z*-scores in the whole spectral region (200–2300 cm^–1^), as reported previously.^21^

#### UV–vis Absorption Spectroscopy

UV–vis absorption spectra of all the samples were obtained using a Cary 60 UV–vis spectrophotometer (Agilent Technologies, Santa Clara, California, USA) in the range of 200–700 nm. The samples were kept in a quartz cuvette of 1 cm path length. All UV–vis data were collected at room temperature.

#### Raman Imaging

RI was performed using a WITec confocal CRM alpha 300 Raman microscope (WITec GMBH, Ulm, Germany). The spectrometer was equipped with an air-cooled solid-state laser operating at 488 nm, with the power in the focus spot equal to 100 μW, and an Andor Newton 970 CCD detector (Oxford Instruments, Abingdon, England) cooled to −60 °C. The laser was coupled to the microscope by an optical fiber with a diameter of 50 μm. A water-immersive Nikon Fluor (60×/1.00 W) objective was used. Spectral resolution was equal to 3 cm^–1^. The monochromator of the spectrometer was calibrated with the use of the radiation spectrum from the calibrated xenon lamp (WITec UV light source). Moreover, the standard alignment procedure (a single-point calibration) was performed before each measurement with the use of the Raman scattering line produced by a silicon plate (520.5 cm^–1^). Raman measurements and data analysis were performed using WITec software (WITec Project Plus 2.10) and OriginPro 2018. All the average Raman spectra were postprocessed (cosmic spike removal with the median filter 5 × 5, smoothing (5–9), and background subtraction with the asymmetric least-squares method) and normalized using *z*-scores in the whole spectral region (200–4000 cm^–1^). The cluster analysis (CA) presented in this work was carried out after cosmic spike removal and background subtraction in the Manhattan distance formulation.

#### Peak Fitting Procedure

The ν(Fe–CN) stretching or δ(Fe–CN) bending modes has very low intensities in the absolute rR spectra and overlaps with the heme modes, making it difficult to obtain the correct frequencies of these modes. We employed naturally abundant CN and its isotopic analogues, namely, the ^13^CN^–^, C^15^N^–^, and ^13^C^15^N^–^ isotopes, to correctly determine the frequencies of the modes associated with the Fe–C≡N fragment, as well as their isotopic shifts. While the difference traces produced clear CN^–^ isotope patterns, the isotopic shifts in the low-frequency region are significantly smaller than the bandwidths of the associated modes. To determine the frequencies of the ν(Fe–CN) stretching mode and the δ(Fe–CN) bending mode, as well as the values of their isotopic shifts, the deconvolution procedure had to be employed. We used a peak fitting procedure provided by OriginPro 2018 and CompareVOA (BioTools Inc., Jupiter, Florida, USA) software, and the fitting was performed employing the Lorentzian function. The number of peaks was restricted only to the modes associated with ν(Fe–CN) or δ(Fe–CN). The intensities and frequencies of the peaks were allowed to change during the iteration cycles, whereas the bandwidth was fixed at 12 cm^–1^. This bandwidth was chosen based on the unrestricted fitting of strong and isolated modes in the absolute spectra of HbCN, such as the ν_7_ mode. The details of the peak fitting procedure were previously published and are described in detail elsewhere.^29^

## Results and Discussion

### UV–vis Electronic Absorption Spectroscopy of HbCN Adducts

**Figure 1:**
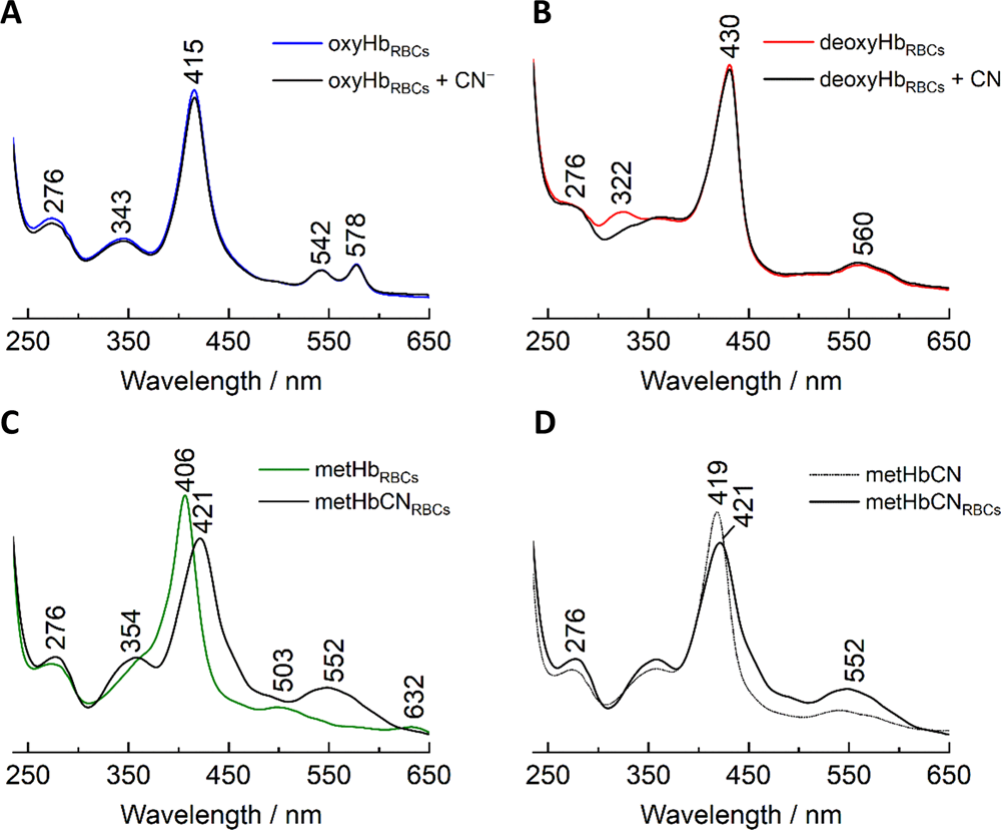
UV–vis absorption spectra of RBCs rich in oxyHb (A), deoxyHb (B), and metHb (C) treated with cyanide ions and a comparison of HbCN formed as an isolated protein with adducts enclosed within RBCs (D). OxyHb (A) and deoxyHb (B) samples were treated with 100-fold molar excess, and (C,D) the metHb sample was treated with 10-fold molar cyanide excess.

The binding of CN^–^ by different forms of hemoglobin was studied using electronic absorption spectroscopy. The UV–vis absorption spectra of oxyHb and deoxyHb (Figure 1A,B, respectively) within RBCs exhibited the Soret bands at 415 and 430 nm, respectively. The addition of 100-fold molar excess of CN^–^ to these samples did not affect the spectra, indicating the extremely low affinity of cyanides to these Hb species, which correlates with the high dissociation constant of cyanide from ferrous Hb estimated by Brunori *et al.* to be around 1 M.^30^ In contrast, the dissociation constant of cyanide from ferric hemes has been estimated to be around 10^–5^ M or lower.^31^ Therefore, the addition of 10-fold molar excess of CN^–^ over heme to the RBCs rich in metHb (Figure 1C) resulted in the immediate formation of HbCN, as indicated by the shift of the Soret band from 406 nm, characteristic for ferric Hb, to about 421 nm, and the appearance of a single Q band at 552 nm.^6,11,32^ Increasing the concentration of cyanides (up to 200-fold molar excess) did not lead to any further spectral changes (data not shown). Similar UV–vis response to the addition of CN^–^ was observed in the experiment performed using isolated metHb (Figure 1D), where the treatment with 10-fold molar excess over the heme of CN^–^ resulted in the clear formation of HbCN adduct, with the Soret band located at around 419 nm slightly red-shifted compared to that of the RBC samples. The UV–vis spectra of HbCN isotopic derivatives (Hb^13^CN, HbC^15^N, and Hb^13^C^15^N) are presented in Figure S1 in the Supporting Information and were characterized by the absence of any differences compared to the spectrum of HbCN itself.

The electronic absorption data provide convincing evidence that the cyanide ions do not effectively bind to the oxy/CO adducts of hemoglobin. In fact, oxyHb must first be chemically (or enzymatically) oxidized to its ferric form to effectively bind CN^–^ ions. Although in living RBCs, human Hb exists mainly in its oxy form and the metHb accounts only for approximately 1% of the whole Hb content in healthy conditions,^10,33,34^ cyanide poisoning leads to the reaction of CN^–^ with a very small fraction of human Hb. Such hemoglobin CN adducts formed are unable to bind and transport oxygen molecules similar to naturally occurring metHb, that is, this physiologically nonfunctional Hb is not more toxic than metHb itself.^9^ Actually, the main target of CN^–^ during cyanide poisoning is another ferric heme protein and one of the major respiratory chain enzyme—cytochrome oxidase a_3_—whose blockage leads to an impairment of oxidative phosphorylation within the mitochondria and in consequence cytotoxic anoxia.^9,35^

### RI of RBCs Containing HbCN Adduct

**Figure 2:**
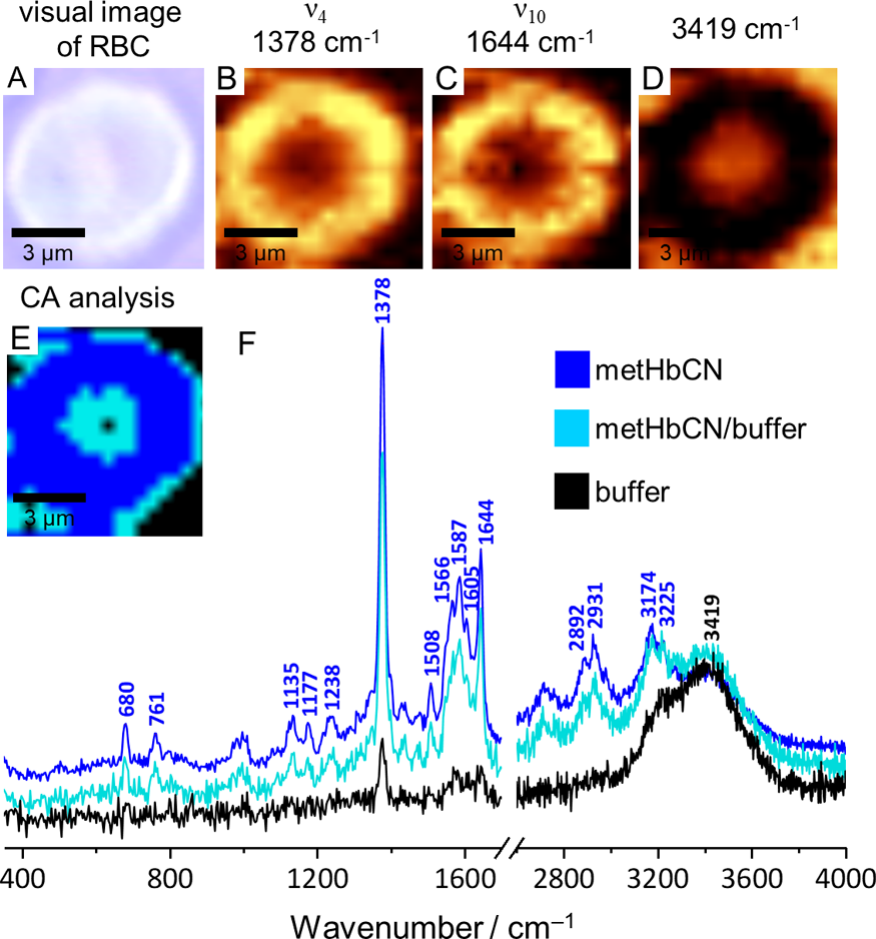
Visual image (×60) of the RBC sample with Raman images, based on the integrations of ν_4_ (1378 cm^–1^) and ν_10_ (1644 cm^–1^) and the solution region (3419 cm^–1^), with the CA image and the corresponding averaged Raman spectra.

The development of confocal microscopy and its combination with Raman spectroscopy allows RI of biological samples, such as RBCs, with high resolution.^24,36^Figure 2 shows an example of the Raman image of a single RBC pretreated with NaNO_2_ (10 mM) and later exposed to KCN (100-fold molar excess). Although the excitation line used in the RI experiment was 488 nm, away from the Soret band maximum of the CN^–^ adducts, the intense ν_4_ and ν_10_ heme modes were strongly enhanced, allowing effective monitoring of the distribution of the Hb molecules. As can be seen on the visual image (Figure 2A), despite the apparently toxic environment of the 100-fold CN^–^ molar excess over the heme concentration, the RBC exhibits normal membrane integrity as well as the functional shape and size of the cell. Moreover, neither the presence of Heinz bodies nor denatured Hb inclusions is observed, indicating that HbCN is not more toxic than metHb. The Raman integration images, presented in Figure 2B,C, were constructed based on the integration of ν_4_ and ν_10_ modes located at 1378 and 1644 cm^–1^, respectively, and show that HbCN adducts are formed solely inside RBCs. The band located at 3419 cm^–1^ originates from ν(O–H); therefore, the image presented in panel D shows the distribution of the surrounding buffer solution and further confirms that the RBC membrane remained intact. The Raman *K*-means CA image presented in Figure 2E reveals chemically similar areas, which were chosen based on the similarities in the collected Raman spectra color-coded as follows: blue—HbCN, black—buffer solution, and cyan—mixture of HbCN and buffer solution. The average Raman spectra acquired from each of the mentioned class are presented in Figure 2F with the corresponding color coding. Altogether, the Raman integration images based on the intensities of the ν_4_ and ν_10_ modes, together with the CA image, show that the HbCN adducts are present exclusively inside the RBCs and that there are no detectable amounts of protein in the surrounding buffer solution, that is, the RBCs exposed to high concentrations of CN salt remain intact.

### rR Spectra of HbCN Adducts

The rR spectra of RBC samples containing hemoglobin CN adducts were compared with that of isolated HbCN protein. The samples were first oxidized, as described in the Materials and Methods section above, and then exposed to a 10-fold molar excess of cyanide ions. The spectra were recorded in the 200–2300 cm^–1^ range. In order to investigate the properties of the Fe–C–N fragments and differences in their geometries, the rR spectra were measured in the presence of natural-abundance CN^–^, as well as in the presence of ^13^CN^–^, C^15^N^–^, and ^13^C^15^N^–^ isotope substituents.

Cyanide is a strong field ligand with a high affinity to ferric Hb; thus, HbCN adducts are characterized by high stability and a population of LS ferric heme.^6,37,38^ The high-frequency rR spectra of RBC containing HbCN adducts and CN^–^ isotopes are shown in Figure S2 in Supporting Information, panel A. The 406.7 nm excitation line used for rR enhancement was in close proximity to the maximum of the Soret band of the HbCN adducts. The most dominant rR band, the ν_4_ mode, is seen at 1378 cm^–1^ and is characteristic of the ferric oxidation state.^4,39^ The spin-state marker modes, ν_3_, ν_2_, and ν_10_, at 1509, 1587, and 1644 cm^–1^, respectively, are typical for low-spin heme species.^4,23,40^ As expected, the CN–^13^CN, CN–C^15^N, and CN–^13^C^15^N difference traces did not reveal the CN^–^-sensitive modes in this region. The corresponding rR spectra were measured for the samples of isolated HbCN protein (figure SM 2, panel B), and no differences were observed between the spectra of free protein and Hb molecules enclosed inside RBCs. It is noted that these rR data are in good agreement with previous rR studies on isolated Hb protein.^1,5,12^

**Figure 3:**
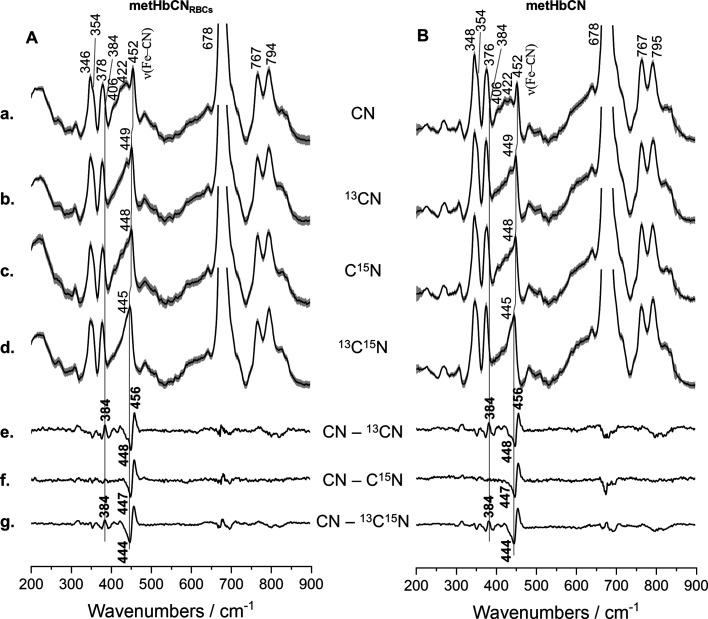
rR spectra of RBCs rich in metHb (A) and isolated metHb (B) treated with potassium cyanide (a) and its isotopic analogues (K^13^CN—b, KC^15^N—c, and K^13^C^15^N—d) in 10-fold molar excess relative to the heme concentration. Spectra were recorded using the 406.7 nm excitation line with power at the sample set approximately to 5 mW and presented in a low wavenumber region (200–900 cm^–1^) with appropriate difference patterns (e–g). All spectra were averaged from three independent experiments from nine single spectra in total (three spectra per experiment). Acquisition time was equal to 5 min per spectrum (10 s and 30 accumulations). All averaged RR spectra are presented with their standard deviation.

Figure 3 shows the rR spectra in the low-frequency region of the HbCN sample inside RBCs, its ^13^CN, C^15^N, and ^13^C^15^N isotopic analogues, and the CN–^13^CN, CN–C^15^N, and CN–^13^C^15^N difference traces (Panel A). The rR spectra are normalized to the dominant ν_7_ mode located at 678 cm^–1^. The ν(Fe–CN) stretching mode is seen around 452 cm^–1^, in a region where this mode is typically observed in histidine-ligated heme proteins.^1,12,13,41^ The rR spectra of ^13^CN-, C^15^N-, and ^13^C^15^N-labeled samples reveal a monotonic downshift of the ν(Fe–CN) stretching mode. The monotonic downshift is further confirmed by the CN–^13^CN, CN–C^15^N, and CN–^13^C^15^N difference traces that are shown in Figure 3e, f, and g, respectively. The monotonic decrease in the ν(Fe–CN) frequencies when the total mass of CN is increased as CN, ^13^CN, C^15^N, and ^13^C^15^N is typically associated with the adoption of the Fe–C≡N fragment’s tilted or “essentially linear” conformation. This nearly linear geometry is characterized by the slightly tilted conformation of the Fe–C≡N linkage relative to the heme normal, which is perpendicular to the heme macrocycle plane. Such distortions are typically induced by steric or electronic factors present in the heme active site and usually result in the activation of the δ(Fe–CN) bending mode.

A careful inspection of the difference traces revealed several additional difference patterns with lower intensities in the region between 300 and 450 cm^–1^. The most pronounced difference is seen at 384 cm^–1^ in the CN–^13^CN and CN–^13^C^15^N traces. The positive and negative difference peaks are especially pronounced in the CN–^13^CN and CN–^13^C^15^N traces, meaning that the mass of carbon has a much more significant effect on Fe–C≡N-associated vibrations than the mass of nitrogen.^12^ The frequency and the value of the isotopic shift of this mode further warrant its assignment to the δ(Fe–CN) bending mode. The additional smaller difference patterns observed in this region arise probably from the vibrational coupling of the δ(Fe–CN) bending mode with the in-plane porphyrin skeletal modes of *E*_u_ geometry, as reported previously.^12,15^

In order to evaluate the differences in the active site geometry between Hb enclosed within a living RBC and that of the isolated protein, the rR spectra of the CN adducts of hemoglobin extracted from the RBC were studied. The low-frequency region of the rR spectra of HbCN and its isotopic analogues are displayed in Figure 3, panel B. The spectra exhibit spectral patterns that are almost identical to HbCN adducts inside RBC, with only minor differences in the relative intensities of modes but no changes in peak frequencies. These differences arise probably from small background changes in the samples inside of the RBC due to the presence of the higher content of the lipid bilayer in the RBC samples. Most importantly, the CN–^13^CN, CN–C^15^N, and CN–^13^C^15^N difference traces reveal that the frequencies and the apparent isotopic shifts of modes associated with the Fe–C≡N unit are very similar to those observed in the spectra of HbCN enclosed in the living cells. It is noted that the rR spectra of both HbCN forms, isolated and RBC-enclosed, resemble the rR spectra of previously published protein quite closely. The frequencies of the heme modes and the isotope-sensitive modes are summarized in Table 1.

**Table 1:** Wavenumbers (cm^–1^) of the Most Prominent Raman Bands with Assignments and Local Coordinates for HbCN Adducts within RBCs and Isolated HbCN Protein^12,41−46^^a^

		wavenumber/cm^–1^	
band	local coordinate	HbCN RBCs	HbCN-isolated Hb
ν_8_		346_m_	346_m_
ν_50_		354_sh_	354_sh_
COO^–^	δ(C_β_C_c_C_d_)	378_m_	376_m_
	δ(Fe–CN)	384_sh_	384_sh_
4-vinyl	δ(C_β_C_a_C_b_)	406_w_	406_w_
2-vinyl	δ(C_β_C_a_C_b_)	422_w_	422_w_
	ν(Fe–CN)	454_m_	454_m_
ν_7_	δ(pyr def)_sym_	678_s_	678_s_
ν_15_	ν(pyr br)	767_m_	767_m_
ν_22_	ν(pyr hr)_asym_	1129_s_	1129_s_
ν_30_	ν(pyr hr)_asym_	1173_m_	1174_m_
ν_5_/ν_13_/ν_42_	δ(C_m_H)	1231_m_	1230_m_
ν_4_	ν(pyr half-ring)sym	1378_vs_	1378_vs_
ν_28_	ν(C_α_C_m_)_sym_	1432_m_	1432_m_
ν_3_	ν(C_α_C_m_)_sym_	1509_s_	1508_s_
ν_11_	ν(C_β_C_β_)	1552_m_	1552_m_
ν_2_	ν(C_β_C_β_)	1588_s_	1588_s_
	ν(C=C)	1624_s_	1624_s_
ν_10_	ν(C_α_C_m_)_asym_	1644_m_	1644_m_
	ν_4_ + ν(Fe–CN)	1825_vw_	1825_vw_
	ν(CN)	2123_vw_	2123_vw_

a The mode notation is based on that proposed by Abe *et al.*(42) and Hu *et al.*(41) ν—stretching, δ—bending, def—deformation, br—breathing, hr—half-ring, qr—quarter-ring, as—asymmetric, sym—symmetric, pyr—pyrrole; vw—very weak; v—weak; m—medium; s—strong, vs—very strong; and sh—shoulder.

As shown in Figure 3, there is a seeming discrepancy between the frequencies of isotope-sensitive modes in the absolute spectra and those in the difference traces; for example, the ν(Fe–CN) stretching mode in the absolute spectrum of the natural abundance CN^–^ is seen at 452 cm^–1^ (Figure 3, panel lA, a), whereas the positive peak in the CN–^13^CN difference trace is at 456 cm^–1^ (Figure 3, panel l A, e). Such apparent inconsistency arises from the fact that the isotopic shifts of the CN^–^-sensitive modes are smaller than the average bandwidths of the associated rR modes. More specifically, the ν(Fe–CN) stretching mode should shift by 5.7, 5.8, and 11.1 cm^–1^ for ^13^CN, C^15^N, and ^13^C^15^N, respectively, instead of the observed corresponding shifts of 8, 9, and 12 cm^–1^ (Figure 3, panel A, traces e, f, and g).

**Figure 4:**
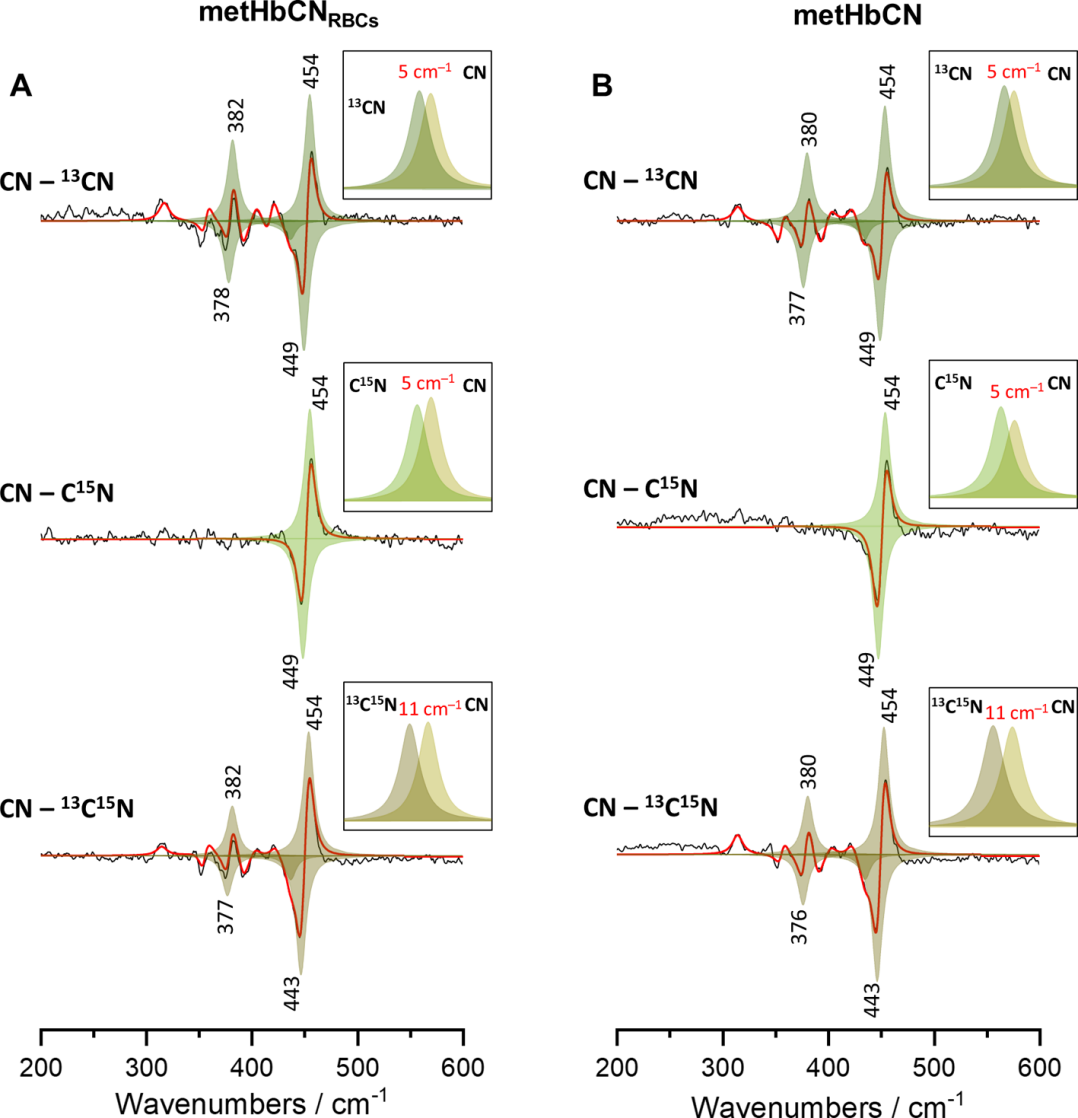
Difference patterns calculated based on the rR difference patterns shown in Figure 3 (black lines) with the simulated traces (red lines) and fitted peaks (shades of green) based on Lorentzian band shape assumption. Panel A corresponds to the spectra presented in Figure 3A obtained for RBCs rich in HbCN adducts, whereas Panel B corresponds to the spectra from Figure 3B obtained for isolated HbCN adducts.

In order to extract the correct frequencies and exact values of the isotopic shifts of these modes, the difference traces were deconvoluted and fitted with appropriate functions. Figure 4 shows the CN–^13^CN, CN–C^15^N, and CN–^13^C^15^N difference traces of HbCN within RBC (panel A) and isolated protein (panel B) that were fitted with peaks based on the Lorentzian band shape. The simulated traces resemble very well the experimental difference traces, allowing to obtain the actual isotopic shifts. The calculated isotopic shifts are now in good agreement with the theoretical values obtained based on the diatomic oscillator approximation. While both ν(Fe–CN) frequencies in the HbCN enclosed in RBC and isolated proteins are now clearly at 454 cm^–1^, the isotopic shifts of ν(Fe–CN) inside RBCs are slightly smaller (1 cm^–1^) compared to the adducts of isolated Hb protein. Furthermore, the small difference patterns present below 450 cm^–1^ in the CN–^13^CN and CN–^13^C^15^N traces were reliably reproduced using the fitting procedure. The band located at around 357 cm^–1^ was not isotope-sensitive; thus, it has to be associated with the porphyrin vibration and was assigned to the ν_50_ mode (not shown).^12,41^ The extracted frequency of the δ(Fe–CN) mode in the HbCN sample within RBCs was found to be 382 cm^–1^. This mode shifted to 378 and 377 cm^–1^ upon substitution with ^13^CN^–^ and ^13^C^15^N^–^, respectively.

Interestingly, for samples of isolated HbCN, the δ(Fe–CN) mode is now established to be at 380 cm^–1^ and exhibits slightly smaller isotopic shifts of 3 and 4 cm^–1^ for ^13^CN^–^ and ^13^C^15^N^–^ substitutions, respectively. In summary, although both HbCN samples, isolated and RBC-enclosed, exhibit identical frequencies of the ν(Fe–CN) stretching modes, their corresponding δ(Fe–CN) bending modes are at a different frequency and exhibit slightly different values of their isotopic shifts. Such deviations indicate changes in the degree of the distortion of the Fe–C≡N fragment, resulting most probably from the subtle alterations of the heme active site structures of Hb in isolated form and that of Hb enclosed in the living RBC that might arise from higher ordering and tighter packing of Hb protein inside RBCs. One can envision that as the quaternary structure of Hb prevents the sterically unhindered linear configuration of the exogenous ligands along the heme axis,^17^ Hb enclosed within RBCs provide even less space in the heme active site, leading to a higher distortion and tilt of the Fe–C≡N linkage. In fact, a relatively recent work by Wood *et al.*, in which Raman spectra were collected with parallel and perpendicular polarized excitation lines, suggested that the Hb molecules enclosed in RBCs undergo significant ordering^47^ which might result in an altered conformation of the heme prosthetic group. This proposal is in line with earlier studies by Pertuz,^48^ in which the author suggested that the high concentration of Hb in RBC (34%) results in a highly packed, semicrystalline state. The small changes observed here in the active site might also account for the physiologically relevant differences in activity or ligand affinity of Hb molecules within RBCs and in their isolated state.^21,49−51^

**Figure 5:**
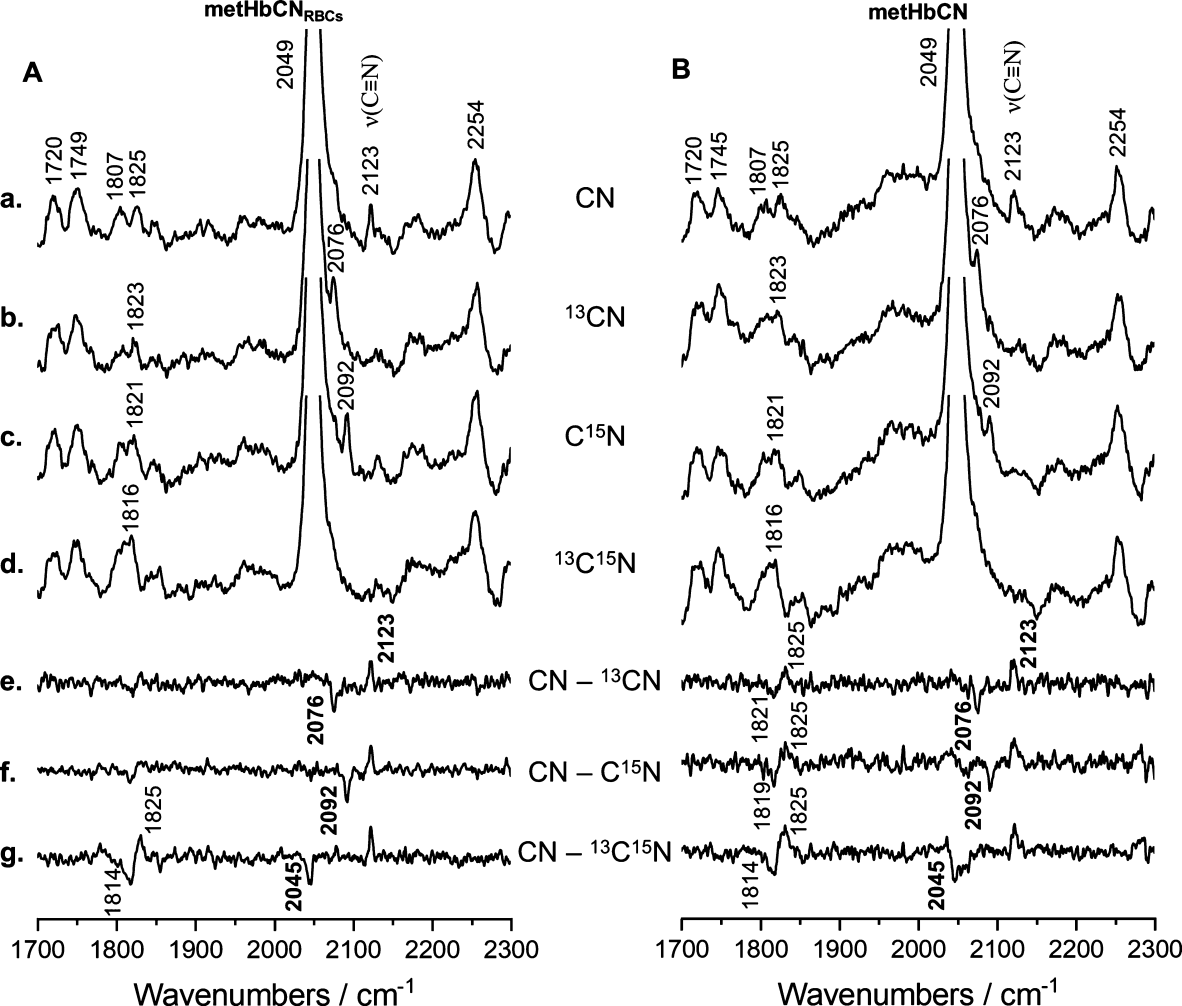
rR spectra of RBCs rich in metHb (A) and isolated metHb (B) treated with potassium cyanide (a) and its isotopic analogues (K^13^CN—b, KC^15^N—c, and K^13^C^15^N—d) in 10-fold molar excess compared to the heme concentration. Spectra were recorded using a 406.7 nm excitation line with power at the sample set approximately to 5 mW and are presented in a high wavenumber region (1700–2300 cm^–1^) with appropriate difference patterns (e–g). All spectra were averaged from three independent experiments from 13 single spectra in total (1/3/9 spectra per experiment). Acquisition time was equal to 5 min per spectrum (20 s and 15 accumulations).

Figure 5 shows the spectral region where the ν(C–N) stretching modes can be typically observed. In both cases, the HbCN enclosed in RBC and isolated protein, the ν(C–N) stretching mode is observed at 2123 cm^–1^. As can be seen from the inspection of difference traces, this mode shifts by 47, 31, and 78 cm^–1^ upon ^13^CN, C^15^N, and ^13^C^15^N substitutions, respectively. This isotopic zig-zag pattern of the ν(C≡N) mode is in agreement with the assignment of the Fe–C≡N conformation to the “essentially linear” or tilted geometry. Interestingly, in this region, another isotope-sensitive line was observed, that is, the mode at around 1825 cm^–1^ shifts approximately 9 cm^–1^ to a lower frequency region upon ^13^C^15^N substitution. We tentatively assign this band to a combination mode of the ν_4_ oxidation state marker and the ν(Fe–CN) stretching mode (1378 + 454 = 1822 cm^–1^). Although the observed 1825 cm^–1^ mode is seen at a higher frequency than it is expected for a ν_4_ + ν(Fe–CN) combination mode, its apparent upshift arises at relatively small isotopic shifts as compared to the bandwidths of the Raman bands. Consequently, the peaks are seen at a higher frequency and exhibit larger-than-expected isotopic shifts, *vide supra*. The spectral parameters of the isotope-sensitive modes for HbCN adducts within RBC and for isolated proteins are summarized in Table 2.

**Table 2:** Wavenumbers (cm^–1^) for the Isotope-Sensitive Modes of HbCN Adducts

	mode	CN	^13^CN	C^15^N	^13^C^15^N
HbCN RBCs	δ(Fe–CN)	382	378		377
	ν(Fe–CN)	454	449	449	443
	ν_4_ + ν(Fe–CN)	1825	1823	1821	1816
	ν(C≡N)	2123	2076	2092	2045
HbCN isolated Hb	δ(Fe–CN)	380	377		376
	ν(Fe–CN)	454	449	449	443
	ν_4_ + ν(Fe–CN)	1825	1823	1821	1816
	ν(C≡N)	2123	2076	2092	2045

## Conclusions

The formation of HbCN adducts inside living functional RBCs was confirmed using RI, which shows that the presence of high concentrations of CN^–^ does not cause any morphological changes to the studied RBCs. UV–vis absorption spectroscopy showed clearly that the CN^–^ ions bind only to the ferric Hb and do not react with either oxyHb or deoxyHb species inside functional RBCs. The rR studies of HbCN adducts and its isotopic analogues (^13^CN^–^, C^15^N^–^, and ^13^C^15^N^–^) allowed for the first time clear characterization of the vibrational modes associated with the Fe–C≡N fragment inside living cells. The data were compared with the spectra of isolated proteins. Deconvolution of the spectral data allowed deciphering correct frequency values of the ν(Fe–CN) stretching and δ(Fe–CN) bending modes. It was found that the ν(Fe–CN) modes have the same frequencies in both the isolated and RBC-enclosed hemoglobin molecules. However, small differences were observed in the frequencies and values of the isotopic shifts of the δ(Fe–CN) bending modes. The data indicate that although the overall tilted conformation of the isolated HbCN is preserved in HbCN enclosed within living cells, there is a small difference in the degree of the axial distortion of the Fe–C≡N fragment, which might arise from the high ordering and tight packing of Hb molecules inside RBCs.

## Supporting Information Available

UV–vis absorption spectra of HbCN isotopic derivatives and rR spectra of RBCs rich in metHb and isolated metHb treated with potassium cyanide and its isotopic analogues in 10-fold molar excess compared to the heme concentration (PDF)

## Abbreviations

rR: resonance Raman
RBCs: red blood cells
Hb: hemoglobin
CN: cyanide ion
HbCN: cyanohemoglobin
CcO: cytochrome *c* oxidase
LPO: lactoperoxidase
MPO: myeloperoxidase
CA: cluster analysis
LS: low-spin
metHb: methemoglobin
oxyHb: oxyhemoglobin
CO: carbon monoxide
Hct: hematocrit
