# Supplementary material for: Probing Heme Active Sites of Hemoglobin in Functional Red Blood Cells Using Resonance Raman Spectroscopy
Source: J Phys Chem B. 2021 Mar 31;125(14):3556–65. doi: 10.1021/acs.jpcb.1c01199 (PMC8154613; doi:10.1021/acs.jpcb.1c01199)
Supplement: Supplementary file 1 — jp1c01199_si_001.pdf [file jp1c01199_si_001.pdf]

Supplemental Information

Probing heme active site of hemoglobin in functional red blood cells using resonance Raman spectroscopy

J. Dybas[a,b*], T. Chiura[a], K. M. Marzec[b], P. J. Mak[a*]

[a] *Saint Louis University, Chemistry Department, 3501 Laclede Ave., 63103 Saint Louis, Missouri, United States*

[b] *Jagiellonian University, Jagiellonian Centre for Experimental Therapeutics (JCET), 14 Bobrzyńskiego Str., 30–348 Krakow, Poland*

This material includes:

Figure S1.

Figure S2.

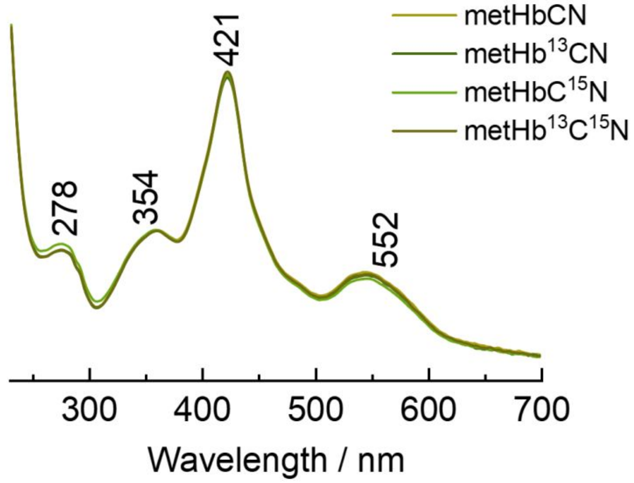

Fig. S1. The UV-Vis absorption spectra of HbCN isotopic derivatives (Hb^{13}CN, HbC^{15}N and Hb^{13}C^{15}N).

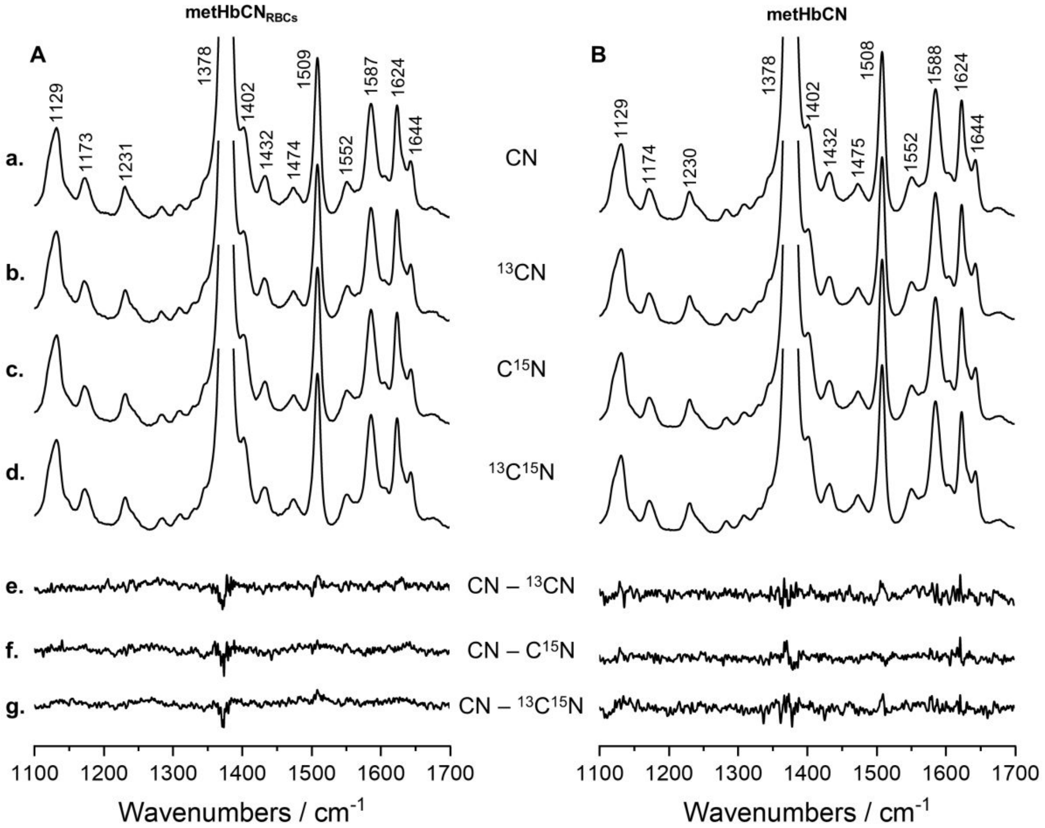

Fig. S2. rR spectra of RBCs rich in metHb (A) and isolated metHb (B) treated with potassium cyanide (a) and its isotopic analogues (K^{13}CN – b, KC^{15}N – c, K^{13}C^{15}N – d) in 10-fold molar excess compared to the heme concentration. Spectra were recorded using 406.7 nm excitation line with power at the sample set approximately on 5 mW and are presented in middle wavenumber region (1100 – 1700 cm^{-1}) with appropriate difference patterns (e-g). All spectra were averaged from 3 independent experiments from 3 single spectra in total (1 spectrum per experiment). Acquisition time was equal to 5 min per spectrum (10 s and 30 accumulations).
